# Public awareness and understanding of stem cell treatments available in Saudi Arabia and their trust in hospitals and research centers involved in stem cell research—a cross sectional study

**DOI:** 10.3389/fpubh.2024.1364809

**Published:** 2024-04-02

**Authors:** Doaa Aboalola, Majed Ramadan, Moayad Baadhaim, Rawiah Alsiary, Heba Badraiq, Tariq Alghamdi, Samer Zakri, Neda Aboulola, Tark Falatah, Dalal Malibari

**Affiliations:** ^1^King Abdullah International Medical Research Center, Jeddah, Saudi Arabia; ^2^King Saud bin Abdulaziz University for Health Sciences, Jeddah, Saudi Arabia; ^3^Ministry of National Guard Health Affairs, Jeddah, Saudi Arabia; ^4^King AbdulAziz University Hospital, Jeddah, Saudi Arabia

**Keywords:** stem cell treatments, stem cell research, misleading stem cell information, public awareness, public health, knowledge, attitude, Saudi Arabia

## Abstract

**Introduction:**

Although stem cell research and therapeutic applications hold great promise for medical advancements, and have rapidly progressed globally, there remains a lack of genuine public awareness of the status of this subject in Saudi Arabia. Successful integration of stem cell therapy into healthcare relies on public awareness, understanding, and trust. Therefore, we aimed in this cross-sectional study to assess the public’s knowledge, awareness, trust, support, participation, and confidence in stem cell treatments and centers involved in it.

**Materials and methods:**

A voluntary questionnaire of 20 questions was distributed randomly via social media outlets.

**Results:**

Three thousand five hundred eighty four individuals participated in the survey, with approximately half of them falling within the age range of 35–50 years (46.71%). Majority of the participants, 90.71%, would like to know more about stem cell therapy and more than half of the participants (56.94%) were unfamiliar with the idea, and a comparable proportion (50.41%) expressed concerns about the safety of stem cell therapy. A lower level of awareness, indicated by a score of 5, was evenly distributed across all age groups and genders. However, regardless of gender, older participants—especially those 50 years of age or older—tended to report higher levels of confidence, trust, and support than participants in other age groups. Moreover, trust, support, participation, and confidence score for those attained high school or less was statistically significantly lower than those attained master’s or PhD degree. Of the participants, 33.57% had either received stem cell therapy themselves or known someone who had; about 24.07% of them reported that it was a cosmetic type of treatment.

**Conclusion:**

The study emphasizes the persistent need for awareness and educational initiatives to minimize the lack of public awareness and understanding of approved stem cell treatments in Saudi Arabia. It advocates for increased education, transparency, and communication to bridge knowledge gaps and enhance public trust to ensure the understanding of successful treatment.

## 1 Introduction

Stem cells are unique, undifferentiated cells in the human body that have the exceptional ability to self-renew and differentiate into any type of cell (1–6). They are present in almost all-human tissue, in embryos, and in fully developed humans (1–5). There are two major types of stem cells: pluripotent stem cells that can differentiate into any cell type in the body, and multipotent stem cells (adult stem cells) that have a more limited ability to become only a specific type of cell (1–5). Stem cell research is constantly evolving with continuous efforts to understand their characteristics, behavior, and potential applications in medicine.

Even if stem cells appear to be the ideal treatment, a number of risk issues still need to be resolved before stem cells can be widely used. In addition to ethical issues, stem cell therapy faces three significant obstacles that will affect its development (1, 2, 7). The first concern is tumorigenicity, which can occur due to genetic mutation, improper cell patterning, or active reprogramming agents. Stem cells, especially pluripotent stem cells, have higher potential to the formation of tumors wither benign or malignant, due to its high proliferation capacity and its ability to differentiate into any cell type in the body (1–5, 7). Another challenge is Immune rejection, where the body’s immune system recognizes the transplanted cells, regardless of its type, as foreign and attacks them, which poses a significant hurdle to stem cell therapy, particularly when it comes to non-homologous cases (1–5, 7). Heterogeneity is also a problem with stem cell therapy, as individual cell lines differ in numerous ways, including shape, proliferation, gene expression, and differentiation into distinct cell lineages (7). Overall, stem cell therapy is a new revolutionary medicine by curing a variety of illnesses and degenerative ailments, and its popularity is growing with every trial. However, there are still numerous challenges to be addressed before stem cells are extensively used in clinical settings.

Hematopoietic stem cell transplantation (HSCT) is currently the oldest and only approved most effective stem cell therapy for the treatment of hematological disorders (8–11).

HSCT involves the transplant of hematopoietic stem cell (HSCs) from a donor to the recipient to replace the recipient’s defective immune system and blood-forming cells. HSCT is used to treat several hematologic conditions, including leukemia, lymphoma, multiple myeloma, sickle cell anemia, thalassemia, and inherited immunodeficiency. Stem cell therapy started in Saudi Arabia in the 1980s in a non-official capacity using hematopoietic stem cell transplantation to treat blood diseases like leukemia and inherited immunodeficiency (12). Research in this area has increased in recent years, resulting in numerous clinical stem cell studies in Saudi Arabia that are registered with the National Institutes of Health (12). A grand total of 6,184 hematopoietic stem cell transplantations were carried out between 1984 and 2016. Of these, 2,598 hematopoietic stem cell transplantations were performed on pediatric patients, while 3,586 were done on adults (12). Although the majority of transplants were carried out using the same sibling donor, more recently, haploidentical, unrelated, and cord blood donors have also been used for hematopoietic stem cell transplants (12). Hematopoietic stem cell transplantation has a long history of use and has shown to be a safe and effective treatment.

Despite the fact that stem cell research and clinical applications have advanced quickly around the world, true public knowledge of the field’s current state is still missing, as numerous reports have indicated (13–17). In our previous study, published in 2020, we surveyed 851 Saudi residents from different regions and different educational levels (18). After examining the survey, we observed a limited amount of knowledge about the general details and the uses of stem cells making the public vulnerable. Moreover, a remarkable result showed that most of the population are requesting researchers to reach out to the society in order to help increase the level of awareness and facts of stem cells due to their high chance of them trying stem cell treatments (facial/ hair) or products that claim to contain stem cells (18). In our second study, published in 2022, we revealed a worrisome inclination and desire to try stem cell treatments among the citizens in Saudi Arabia, as well as a worrying ignorance and unawareness of the potential detrimental health effects of such unproven treatments (19). Therefore, our previous results emphasize the urgent need for educational initiatives that would enlighten the public on the most recent developments in the field of stem cell research.

In conclusion, Stem cells are cells that have the ability to differentiate into various cell types. They have the potential to replace damaged tissues and organs, making them a promising treatment option for a range of medical conditions. In Saudi Arabia, stem cell therapy is an emerging field that has seen significant progress in recent years. However, despite the availability of approved and legitimate treatments and clinical trials, there is still a lack of public awareness and understanding of these options. Moreover, the level of trust in hospitals and research centers involved in stem cell research is also a concern. This paper will examine these issues by assessing the publics’ awareness and understanding of approved and legit stem cell treatments and clinical trials available in Saudi Arabia and their trust in hospitals and research centers involved in stem cell research. It will also highlight the need for increased public education and outreach efforts.

## 2 Materials and methods

### 2.1 Study design, setting, and participants

This exploratory study employed a cross-sectional survey design aimed to investigate knowledge, trust, support, participation, and confidence in stem cells treatments among individuals in Saudi Arabia aged 18 years and older. Ethical approval was obtained from the local research ethics committee (IRB) at King Abdullah International Medical Research Center (IRBC/0995/23). A Google form with 20 voluntary questions was randomly sent to the general population via social media platforms. Before completing the questionnaire, each participant acknowledged their agreement to participate by accepting the informed consent form.

Since not all members of the public use social media, the survey’s results from the general population may be biased; but, given the large number of replies, this problem may be considered minimum.

### 2.2 Sample size, pilot test

The minimum required sample size determined to achieve adequate statistical power and precision was calculated based on metrics specific to our target population. SAS 9.4 was utilized to compute the sample size with a confidence level of 95% and a margin of error of 5%. However, our researchers aimed to collect samples from diverse regions within the Kingdom of Saudi Arabia, considering the potential variation in stem cell knowledge across the nation’s 13 regions. The study employed a convenience sampling method, where participants were selected based on their availability and willingness to participate. To ensure the clarity and comprehensibility of the questionnaire and validate its questions, a pilot test involving 20 participants was conducted. Subsequent to receiving feedback from these participants, we made revisions to unclear questions and shortened lengthy ones. Following these modifications, the final version of the survey was distributed to the general public.

### 2.3 Variables

**Figure 1:**
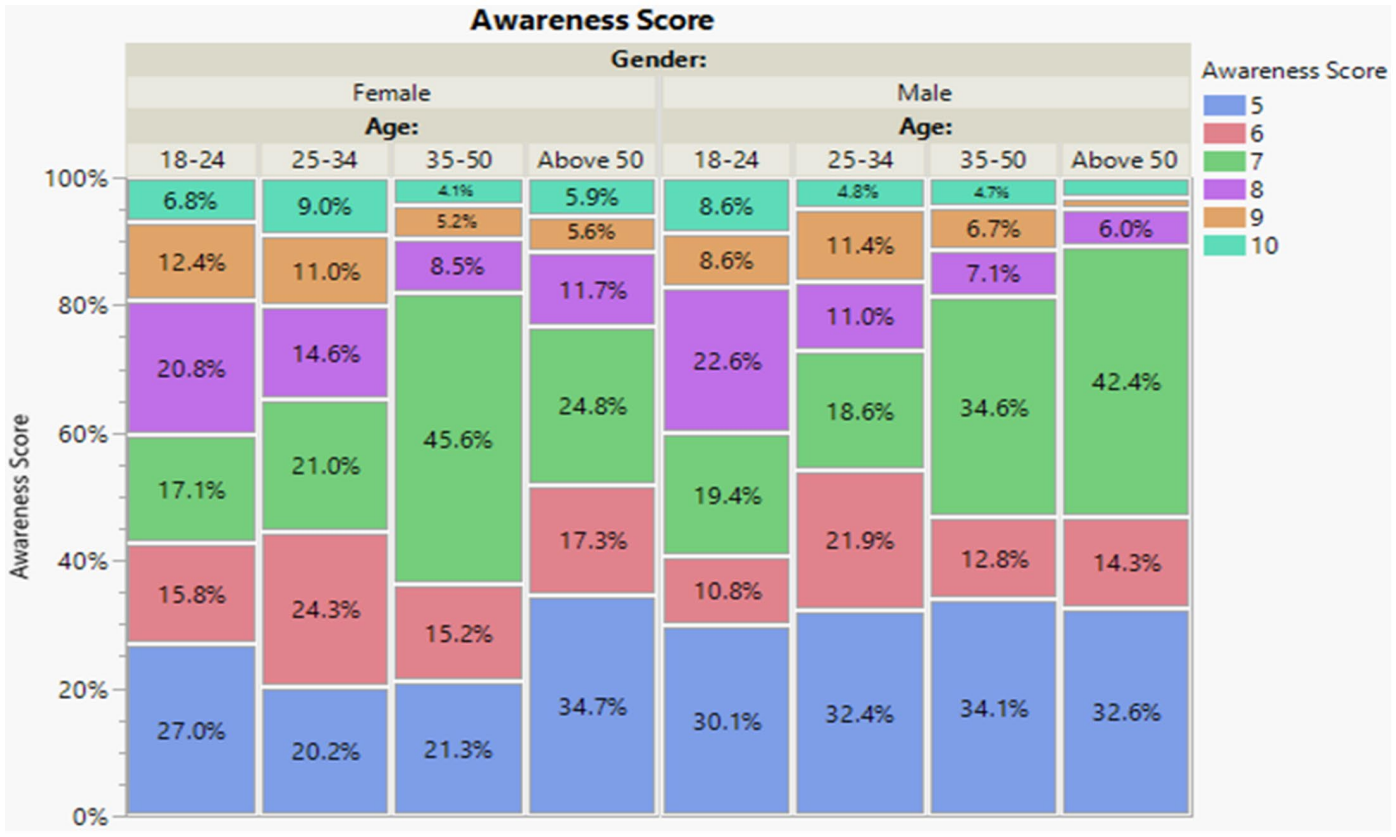
Awareness score by age and gender. Level of awareness in both genders within different ages is moderate. At age 35–50 and above 50 demonstrated a higher level of awareness in comparison to the younger age group. The lower level of awareness, indicated by a score of 5, ranged from 20.2 to 34.7% and was pretty evenly distributed across all age groups and genders.

**Figure 2:**
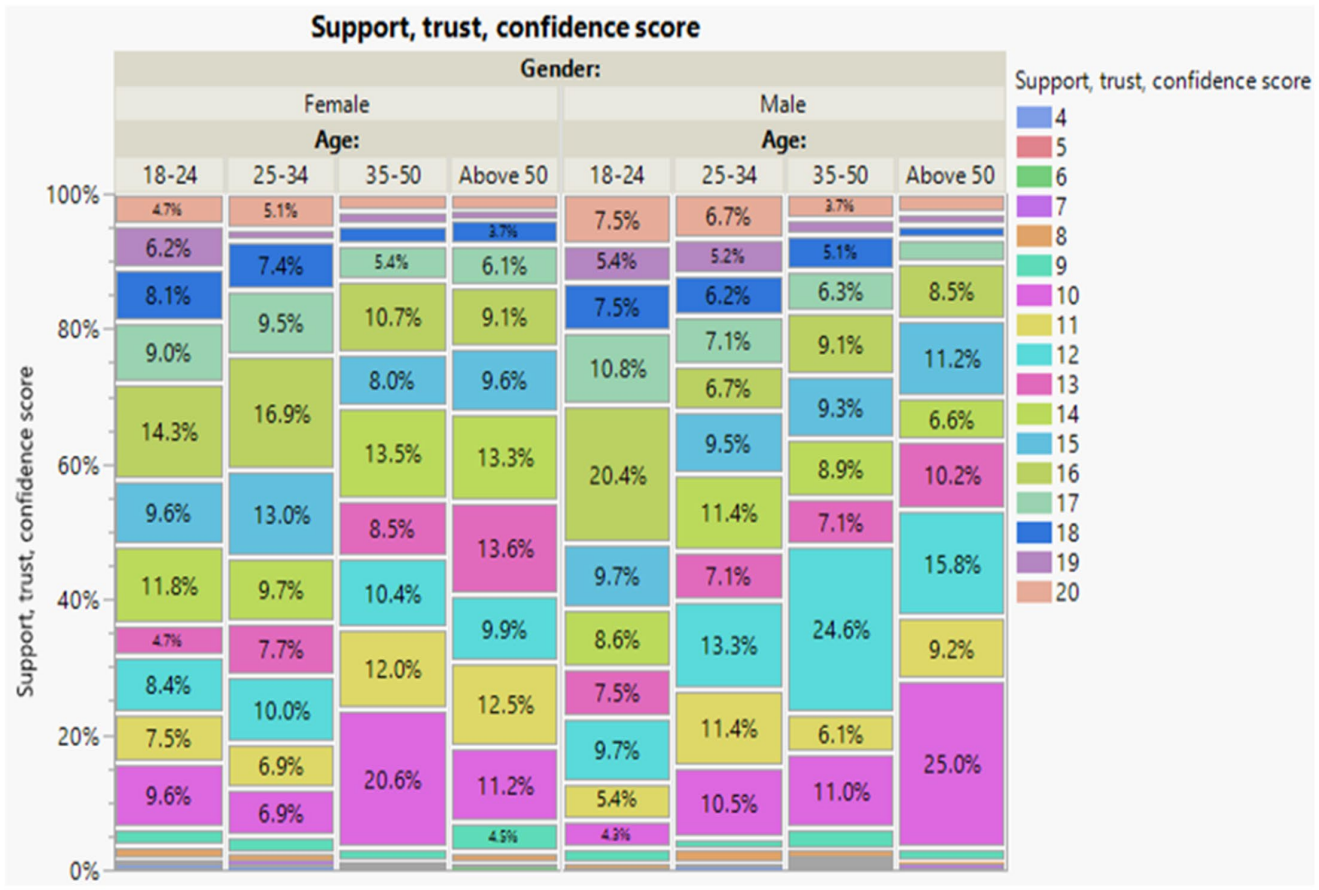
Support, trust, and confidence score by age and gender. The percentage distribution values that signify the proportion of participants within different categories. The overall trend reveals that older groups report higher levels of support, trust, participation and confidence.

The primary outcomes of the study were first awareness, second a combined score of trust, support, participation, and confidence. Outcomes were scored based on the Likert scale questions used in the survey. The awareness score was scored based on the six questions under the awareness domain ranging from a minimum of five points to a maximum of 10 score points (Figure 1). Also, trust, support, participation, and confidence score were scored based on six questions under the domain of trust, support, participation, and confidence ranging from a minimum of five points to a maximum of 20 points (Figure 2). For both outcomes, increasing scores indicate higher levels of awareness, trust, support, participation, and confidence. Our independent variables included demographics: age, gender, and education attainment; and stem cells related questions: have you or anyone you know ever received a stem cell treatment?, type of treatment? have you or anyone you know ever received a stem cell treatment, Would you like to know more about stem cell therapy and its uses?, How familiar are you with the concept of stem cells?, and How safe do you believe stem cell treatments to be?

### 2.4 Statistical analysis

Prior to the statistical analysis, raw data was gathered and checked for any errors or information loss.

In the initial analysis, participant characteristics were assessed using chi-square tests for categorical variables and *t*-tests for normally distributed numeric variables. Furthermore, we assessed the internal consistency or reliability of the measurements through the Cronbach’s alpha test. The Cronbach’s alpha test yielded a result of 83%, indicating a satisfied level of reliability. Subsequently, in the multivariate analysis aimed at investigating the relationships between awareness scores and trust, support, participation, and confidence scores, considering demographic factors, two generalized linear regression models were employed. These models were rigorously evaluated to ensure they met key assumptions, including linearity, homoscedasticity (constant variance), normality, absence or low multicollinearity, and independence, all of which were found to be satisfied. Significance in the multivariate analyses was determined at a threshold of *p* < 0.05. All statistical analyses were conducted using SAS 9.4.

## 3 Results

### 3.1 Demographic and characteristics of the participants

The study enrolled a total of 3,584 participants, with approximately half of them falling within the age range of 35–50 years (46.71%). The majority of participants identified as female (63.34%), and over half of the cohort had attained at least a diploma or bachelor’s degree (56.53%). About a third of the overall population (33.57%) either had personal experience with stem cell treatment or knew someone who had undergone such treatment. Additionally, around a quarter of the participants (24.07%) had received a cosmetic type of treatment, followed by cancer treatment, which was reported by 5.8% of the participants. The study’s findings revealed that the mean awareness score was 6.28, with a median score of 5. Regarding trust, support, participation, and confidence, the mean score was 13.49, and the median score was 13, indicating a fairly consistent distribution of responses across these categories. A substantial proportion of the participants (90.71%) expressed a desire to learn more about stem cell therapy. However, over half of the participants reported being unfamiliar with the concept (56.94%), and a similar percentage expressed concerns about the safety of stem cell therapy (50.41%) (Table 1).

**Table 1:** Demographic characteristics of the participants (*N* = 3,584).

Age	*N* (%)^a^
18–24	415 (11.58)
25–34	601 (16.77)
35–50	1,674 (46.71)
50>	894 (24.94)
**Gender**	
Female	2,270 (63.34)
Male	1,314 (36.66)
**Education**	
High school or less	530 (14.79)
Diploma/bachelor	2,026 (56.53)
Master/PhD	992 (27.68)
**Have you or anyone you know ever received a stem cell treatment?**	
Yes	1,203 (33.57)
No	2,381 (66.43)
**Type of stem cell treatment**	
No treatment/or do not know	2,194 (61.21)
Cosmetic	863 (24.07)
Cancer	211 (5.8)
Blood related disorder	116 (3.3)
Other treatment	200 (5.5)
**Awareness score** ^b^	
Mean/median/standard deviation	6.28/5/1.71
**Trust, support, participation, and confidence score**^c^	
Mean/median/standard deviation	13.49/13/3.0
**Would you like to know more about stem cell therapy and its uses?**	
Yes	3,251 (90.71)
No	333 (9.29)
**How familiar are you with the concept of stem cells?**	
Familiar	405 (11.3)
Very familiar	131 (3.65)
Moderate	1,007 (28.09)
Not familiar	2,041 (56.94)
**How safe do you believe stem cell treatments to be?**	
Extremely dangerous	86 (2.4)
Dangerous	192 (5.35)
Not sure	1,807 (50.41)
Safe	1,336 (37.27)
Extremely safe	163 (4.54)

a Sample size and percentage *N* (%).

b Score range min/max (5–10) the higher score the higher the awareness.

c Score range min/max (4–20) the higher score the higher the trust, support, confidence, and willingness to participate.

### 3.2 Awareness score by age, and gender

Both genders, as well as participants within the age groups of 35–50 and those above 50, exhibited a moderate level of awareness, which was notably higher compared to the younger age groups. In contrast, the lower level of awareness, as denoted by a score of 5, was fairly evenly distributed across all age groups and genders, ranging from 20.2 to 34.7% (Figure 1).

### 3.3 Trust, support, participation, and confidence score

The highest levels of support, trust, participation, and confidence, with a score of 7.5%, were observed among the young age group (18–24) males. On the other hand, the median and moderate levels of support, trust, participation, and confidence, all with a score of 13, were notably higher among the older age group (50 or above) males, accounting for 25% of this group. Similarly, older age group (50 or above) females also demonstrated a substantial level of support, trust, and confidence, totaling 20% of age group (35–50). These findings indicate that older participants, particularly those aged 50 or above, tended to report higher levels of support, trust, and confidence compared to other age groups, regardless of gender (Figure 2).

### 3.4 Association between awareness, trust, support, participation, and confidence with demographics

**Figure 3:**
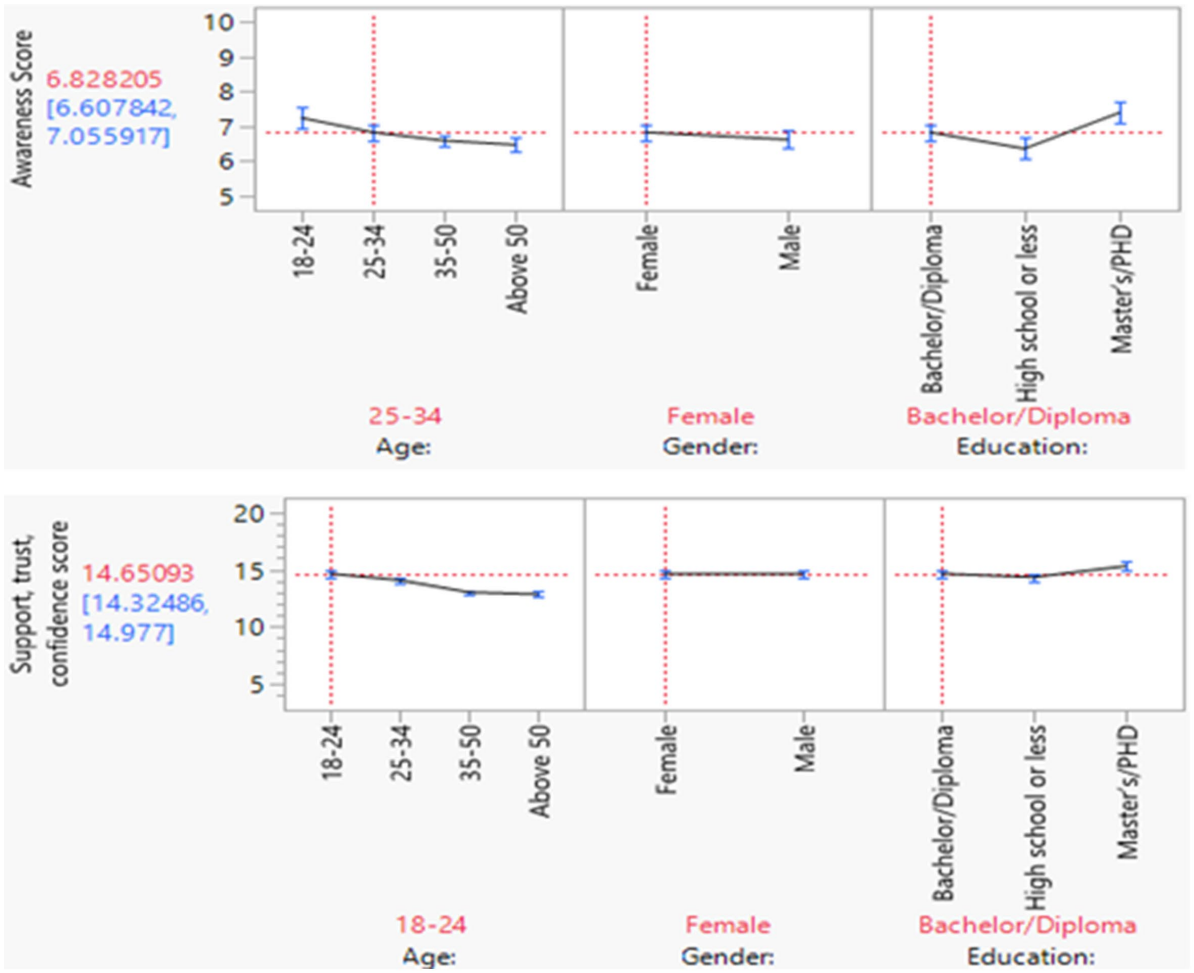
Generalized linear model for awareness, and support, trust, and confidence score. The awareness mean score for female was slightly higher than male. Awareness, trust, support, participation, and confidence mean score for the younger age group was significantly higher compared to the older age groups; but was lower than those who attained master’s or PhD degree.

In the generalized linear regression model, the awareness mean score of the age group 18–24 years old was statistically significantly higher than age group 50 or > [estimate 0.06; Confidence interval (CI) (0.03, 0.09); *p*-value < 0.0001]. However, the awareness mean score of the age group 35–50 was statistically significantly lower than age group 50 or > [estimate −0.02; CI (−0.04, −0.007); *p*-value 0.0006] (Table 2; Figure 3). Moreover, the awareness mean score for female was slightly higher than male [estimate −0.02; CI (0.001, 0.2); *p*-value 0.02]. The awareness mean score for those attained high school or less was statistically significantly lower than those attained master or PhD degree [estimate −0.02; CI (−0.1, −0.04); *p*-value < 0.0001] (Table 2; Figure 3). Similarly in trust, support, participation, and confidence score, younger age group (18–24), and (25–34) have statistically significantly higher mean score than age group 50 or > [estimate 0.99; CI (0.74, 1.24); *p*-value < 0.0001], [estimate 0.43; CI (0.22, 0.63); *p*-value < 0.0001] respectively (Table 3; Figure 3). The trust, support, participation, and confidence score for those attained high school or less was statistically significantly lower than those attained master’s or PhD degree [estimate −0.42; CI (−0.62, −0.22); *p*-value < 0.0001] (Table 3; Figure 3).

**Table 2:** Association between awareness and demographics.

	Awareness score			
	Estimate^d^	SE^c^	95% confidence interval	*p*-value^a^
**Age**				
18–24	0.06	0.02	(0.03, 0.09)	<0.0001
25–34	0.01	0.01	(−0.01, 0.03)	0.57
35–50	−0.02	0.01	(−0.04, −0.007)	0.006
50>	Ref^b^	Ref	Ref	Ref
**Gender**				
Female	0.02	0.006	(0.001, 0.2)	0.02
Male	Ref	Ref	Ref	Ref
**Education**				
High school or less	−0.07	0.01	(−0.1, −0.04)	<0.0001
Diploma/bachelor	−0.003	0.009	(−0.02, 0.01)	0.75
Master/PhD	Ref	Ref	Ref	Ref

a Generalized linear model with Poisson distribution.

b (Ref) reference group.

c SE standard error.

d Mean differences.

**Table 3:** Association between trust, support, participation and confidence with demographics.

	Trust, support, participation, and confidence score			
	Estimate^d^	SE^c^	95% confidence interval	*p*-value^a^
**Age**				
18–24	0.99	0.12	(0.74, 1.24)	<0.0001
25–34	0.43	0.1	(0.22, 0.63)	<0.0001
35–50	−0.62	0.07	(−0.77, 0.46)	<0.0001
50>	Ref^b^	Ref	Ref	Ref
**Gender**				
Female	0.002	0.05	(−0.1, 0.1)	0.96
Male	Ref	Ref	Ref	Ref
**Education**				
High school or less	−0.42	0.1	(−0.62, −0.22)	<0.0001
Diploma/bachelor	−0.12	0.07	(−0.25, 0.01)	0.08
Master/PhD	Ref	Ref	Ref	Ref

a Generalized linear model with normal distribution.

b (Ref) reference group.

c SE standard error.

d Mean differences.

## 4 Discussion

Moreover, in Saudi Arabia, we have two public cord blood storage facilities were subject to a Fatwa (Islamic legal judgment) issued by the Muslim World League’s Jurisprudential Council (15, 20). After the first open cord blood bank was created at King Faisal Specialized Hospital and Research Center in 2006, a second bank was established at the King Abdullah International Medical Research Center (KAIMRC) in 2009 (15). The nonprofit KAIMRC Umbilical Cord Blood Bank provides hematological stem cells to those in need and for use in research (15, 20–22). The National Guard Health Affairs Division developed the Saudi Stem Cell Donor Registry (SSCDR) in 2011 with the aim of enhancing matching. It is the first stem cell donor registry in the Middle East (15, 22). However, the majority of citizens in Saudi Arabia are uninformed of the existence of such reliable facilities and the services they provide (14). Additionally, there has been a concerning spread of false information about stem cell therapies among the population. This calls for a concentrated effort to improve public and medical professional understanding of the condition of stem cell therapy today. We must therefore continue and advance our research and broaden it in order to evaluate public knowledge of and trust in Saudi Arabian hospitals and stem cell research institutions, as well as public awareness and understanding of approved and legitimate stem cell treatments and clinical trials. Our main goal is to contribute to the development of real health beliefs as a baseline and to advance improved behavior and knowledge, which will improve public choices and lessen the likelihood of stem cell tourism and deceptive commercial marketing.

Currently on the market, stem cell cosmetic treatment options use stem cell origin either from plant or from autologous stem cells as a source. The most commonly and widely used commercial stem cell cosmetic products are those derived from plant stem cells for skin and hair care. Hundreds of off-the-shelf products consist of active ingredients from plant stem cell extracts mixed with various compounds and preservatives (23). The marketing of esthetic procedures uses an autologous regeneration procedure that is platelet-rich plasma (PRP), which contains cytokines, chemokines, growth factors, and plasma proteins. Surprisingly, real stem cells do not exist in this type of technique, although it is falsely advertised in the market as a stem cell-based regenerative treatment (23–25). These direct-to-consumer stem cell product lines and PRP have no serious side effects when applied on skin or hair; the only worst circumstance is being a victim false advertising (25).

On the other hand, the unique features of stem cells make up their promises to restore the functions of diseased or damaged tissues and organs. Several reports have demonstrated an increase in “stem cell clinics” that offer unproven stem cell therapies using actual stem cells for both medical and cosmetic conditions, despite the lack of scientific support for their safety and efficacy (26–29). These treatments are extremely dangerous and life threatening.

In spite of the existing evidence on the health benefits of stem cell therapy, there exist challenges that have led to the low uptake of the medical intervention. The findings of this study suggest that gender, age, education levels, awareness levels, and trust are the key determinants of the uptake of stem cell therapy in Saudi Arabia. A total of 3,584 participants were included in the study, 46.71% of them were between the ages of 35 and 50, with females as the majority of participants (63.34%). According to our data, 33.57% of the participants had either personally experienced stem cell therapy or knew someone who had. This finding implies that stem cell therapy is a frequent practice that has attracted much public interest and emphasizes the expanding acceptance and application of this medical strategy in treating a range of health issues. Furthermore, out of the people who had personal experience with stem cell treatment or knew someone who had undergone it, about 24.07% had received a cosmetic type of treatment. This might be attributed to the increasing number of clinics offering cosmetic stem cell treatments, allowing stem cell therapy to gain popularity in the cosmetic field. However, the level of patients and their relatives’ awareness of stem cell uses in Saudi Arabia was notably low, with more than half of the study population reporting unfamiliarity with the concept of stem cell therapy. The findings in this study were congruent with the findings of Eldesouky (30), who reported low awareness of the use of stem cells before an educational intervention. Additionally, Alfotawi and Almuraikhi (31) and Aboalola et al. (19) reported low or moderate patient awareness levels about the detailed use of stem cells. These findings highlight a significant gap in public education efforts regarding regenerative medicine and the intricacies of stem cell therapies.

Additionally, more than half of those surveyed claimed to be unaware of stem cell therapy (56.94%) or worried about its safety (50.41%). The lack of knowledge and safety issues around stem cell therapy emphasizes the necessity to increase stem cell therapy awareness. Since medical experts and students have a better understanding of stem cells and their role in medicine (32, 33), they should provide accurate information to the general public in order to allay worries and enable decision-making regarding their healthcare options. In addition, our statistics also revealed three other facts. First, even though about 45.3% of the respondents said they understood how stem cell therapy is utilized in clinics, more than half of them, 28.4%, answered incorrectly. Second, about 22.1% of the participants were unaware of what a clinical trial is. Third, low level of awareness was evenly distributed across all age groups and genders, ranging from 20.2 to 34.7%. This underlines the necessity for further education, which may be accomplished through awareness campaigns, open forums, and honest discussions that present actual information regarding the positive and negative aspects of stem cell therapy. The complexity of stem cell research and its potential influence on healthcare improvements may also be better understood by encouraging open dialog between scientists, policymakers, and the public.

This study found that patients and their relatives’ education levels predicted knowledge and understanding of stem cell use. Individuals who had attained a master’s degree or higher were more likely to be knowledgeable about stem cell use compared to those who had reached high school or had lesser academic qualifications. These findings agreed with those of AlSubaie et al., who found an association between the education level of an individual and knowledge of stem cell use (34). This can be alluded to as general information available to the public that could be complex for those of lower education levels to decipher. Relatively younger individuals (18–24 years) were more knowledgeable about the uses of stem cells compared to individuals who belonged to older age groups (50 years and older). These findings are in agreement with our previous study (19), which suggested that the high knowledge levels of younger populations were due to their ability to access information on the internet, driven by learning curiosity coupled with evolving societal attitudes.

The level of acceptance for stem cell treatments by patients and their relatives in Saudi Arabia was predicted by knowledge levels, levels of trust, levels of support, and confidence. Poor attitude toward stem cell therapy before an educational intervention was positively associated with low awareness levels, indicating that education influenced the awareness of patients and their relatives, suggesting that education predicted acceptance of stem cell use. It is worth noting that acceptance of cell and gene therapies varied among patients but generally increased after the provision of information (35). Moreover, the acceptance of stem cell treatment by patients and their relatives was based on the trust and confidence they had in the health providers. Studies by previous researchers have shown that healthcare providers are the most trusted to disseminate health education, thereby making them the most convenient and most sought source of information on stem cell treatment (31, 34, 36). Furthermore, the concerns expressed by approximately half of the participants regarding the safety of stem cell therapy should not be overlooked. Addressing the health concerns associated with the medical intervention will likely increase acceptance rates and build the trust and confidence of patients and the public in the safety and efficacy of stem cell therapy.

Lastly, in addition to these key findings, it is noteworthy that participants had various perceptions of the types of diseases or conditions that stem cell therapies address. These perceptions ranged from genetic blood disorders, cancer, and neurological diseases, to cosmetic concerns for skin and hair loss (data not shown). As public awareness and education efforts progress, aligning these perceptions with current scientific understanding will be essential to ensuring informed decision-making and responsible engagement with stem cell treatments. This study, therefore, recommends a scale-up to patients’ and their relatives’ education on stem cell therapy to bridge the knowledge gaps identified in the present study and facilitate informed decision-making to enhance acceptance of the medical practice.

## 5 Conclusion

Our results show a concerning lack of understanding and awareness of the potential harmful health effects of such experimental treatments, as well as a worrisome desire try it. This draws attention to the pressing need for public education initiatives that would enlighten the public about the state of stem cell research today. Furthermore, since patients and their families view health care providers as reliable and trustworthy sources of information, such campaigns must involve them as well.

Despite the fact that stem cell therapy has the potential to completely transform healthcare in Saudi Arabia and around the world, there are still a number of pressing issues that need to be resolved. Increased education, communication, and transparency initiatives are required to address the public’s lack of knowledge of ethical therapies and clinical trials as well as their anxieties and concerns over their safety and regulation. Together, government organizations, medical practitioners, and research institutions can create a more knowledgeable and dependable community and insure that patients have access to trustworthy and safe stem cell therapies.

In conclusion, the lack of public awareness and understanding of approved stem cell treatments in Saudi Arabia is a serious concern. This can contribute to skepticism among members of the public and lead to missed opportunities for patients who could benefit from these treatments. Education and outreach efforts, including the provision of accurate information, education for healthcare providers, and awareness campaigns, can help increase public knowledge and understanding of approved stem cell treatments. Additionally, building trust in the healthcare system through transparency and commitment to patient welfare is essential in encouraging patients to pursue these treatments. Ultimately, a greater understanding of approved stem cell treatments can improve patient outcomes and contribute to the advancement of medical research and treatment in Saudi Arabia.

## Data availability statement

The original contributions presented in the study are included in the article/Supplementary material, further inquiries can be directed to the corresponding author.

## Ethics statement

The studies involving humans were approved by Local research ethics committee (IRB) at King Abdullah International Medical Research Center (IRBC/0995/23). The studies were conducted in accordance with the local legislation and institutional requirements. Written informed consent for participation was not required from the participants or the participants’ legal guardians/next of kin in accordance with the national legislation and institutional requirements.

## Author contributions

DA: Writing – review & editing, Writing – original draft, Visualization, Validation, Supervision, Resources, Project administration, Methodology, Investigation, Data curation, Conceptualization. MR: Writing – review & editing, Writing – original draft, Validation, Software, Methodology, Investigation, Formal analysis, Data curation. MB: Writing – review & editing, Writing – original draft, Validation, Investigation. RA: Writing – review & editing, Writing – original draft, Validation, Investigation. HB: Writing – review & editing, Writing – original draft, Validation, Investigation. TA: Writing – review & editing, Writing – original draft, Validation, Investigation. SZ: Writing – review & editing, Writing – original draft, Validation. NA: Writing – review & editing, Writing – original draft, Investigation. TF: Writing – review & editing, Writing – original draft, Investigation. DM: Writing – review & editing, Writing – original draft.
