# Supplementary material for: Public awareness and understanding of stem cell treatments available in Saudi Arabia and their trust in hospitals and research centers involved in stem cell research—a cross sectional study
Source: Front Public Health. 2024 Apr 2;12:1364809. doi: 10.3389/fpubh.2024.1364809 (PMC11018913; doi:10.3389/fpubh.2024.1364809)
Supplement: Supplementary file 1 [file Data_Sheet_1.docx]

1. **Supplementary material:**

**The questionnaire:**

**- Demographics:**

1. How old are you?

- 18-24
- 25-34
- 35-50
- Above 50

2. Gender:

- Male
- Female

3. Education:

- Did not complete high school
- High school
- Diploma
- Bachelor
- Master’s
- PhD

4. Have you or anyone you know ever received a stem cell treatment?

- Yes
- No
- Not sure

5. If your answer is yes for question 4, what is the treatment for?

- Cosmetics
- Cancer treatment
- Blood related disorder
- Not sure
- Other:______________
- My answer is NO

6. Would you like to know more about stem cell treatments?

- Yes
- No
- Not sure

**- Awareness:**

7. Are you aware of any stem cell treatments currently available in Saudi Arabia?

- Yes
- No
- Not sure

8. If your answer is yes to the previous question, what is the method used for stem cell treatments?

- Stem cell transplantation after extracting it from blood or bone marrow
- Separate it from the blood in the clinic and then inject it directly into the desired location to be treated
- Blood transfusion
- Not sure

• My answer is NO

9. Are you aware of any ongoing stem cell clinical trials in Saudi Arabia, the Middle East, or around the world?

- Yes
- No
- Not sure
- I do not know what is a clinical trial

10. Are you aware of any diseases treated by stem cells?

- Yes
- No
- Not sure

11. If your answer is yes to the previous question, what is the type of disease?

(Type your answer)

12. Are you aware that stem cells can be donated?

- Yes
- No
- Not sure

13. Are you aware about the availability of Stem Cell Donor Registries?

- Yes
- No
- Not sure

14. Do you think that there should be more awareness campaigns and programs regarding stem cells and their applications?

- Yes
- No
- Not sure

**- Trust, support, participation, and confidence:**

15. On a scale of 1-5: How familiar are you with the concept of stem cells?

- 1 (Not familiar)
- 2 (Not sure)
- 3(Moderate)
- 4 (Familiar)
- 5 (Very Familiar)

16. On a scale of 1-5: How safe do you believe stem cell treatments to be?

- 1 (Extremely dangerous)
- 2 (dangerous)
- 3 (Not sure)
- 4 (Safe)
- 5 (Extremely safe)

17. On a scale of 1-5: How strongly do you support/oppose stem cell research and therapy?

- 1 (Strongly oppose)
- 2 (oppose)
- 3 (Not sure)
- 4 (Support)
- 5 (Strongly support)

18. On a scale of 1-5: How much trust do you have in hospitals and research centers involved in stem cell research?

- 1 (Strongly do not trust)
- 2 (I do not trust)
- 3 (Not sure)
- 4 (Trust)
- 5 (Strongly trust)

19. On a scale of 1-5: How confident are you in the accuracy and credibility of the information provided by hospitals and research institutions involved in stem cell research and treatment?

- 1 (Not confidant)
- 2 (Not sure)
- 3(Moderate)
- 4 (Confidant)
- 5 (Very confidant)

20. On a scale of 1-5: How likely are you to participate in a stem cell research study conducted by hospitals or research centers?

- 1 (Un-likely)
- 2 (Not sure)
- 3 (Maybe)
- 4 (Likely)
- 5 (Very likely)
